# Improved treatment satisfaction and weight-related quality of life with exenatide once weekly or twice daily^1^

**DOI:** 10.1111/j.1464-5491.2009.02752.x

**Published:** 2009-07

**Authors:** J H Best, K S Boye, R R Rubin, D Cao, T H Kim, M Peyrot

**Affiliations:** Amylin Pharmaceuticals Inc.San Diego, CA; * Eli Lilly and CompanyIndianapolis, IN; † Johns Hopkins UniversityBaltimore, MD; ‡ Loyola CollegeBaltimore, MD, USA

**Keywords:** exenatide, treatment satisfaction, Type 2 diabetes mellitus

## Abstract

**Aims:**

To assess treatment satisfaction and weight-related quality of life (QOL) in subjects with Type 2 diabetes treated with exenatide once weekly (QW) or twice daily (BID).

**Methods:**

In this 52-week randomized, multi-centre, open-label study, 295 subjects managed with diet and exercise and/or oral glucose-lowering medications received either exenatide QW or BID during weeks 1–30; thereafter, subjects receiving exenatide BID were switched to exenatide QW, with 258 total subjects receiving exenatide QW during weeks 30–52. Diabetes Treatment Satisfaction Questionnaire—status (DTSQ-s) and Impact of Weight on Quality of Life—Lite (IWQOL-Lite) were assessed at baseline and weeks 30 and 52. Mean group changes from baseline to week 30 were estimated by ancova; changes from week 30 to week 52 were assessed by Student’s *t*-test.

**Results:**

Statistically significant improvements from baseline to week 30 were observed in both treatment groups for DTSQ-s and IWQOL-Lite measures, with significantly greater reduction in perceived frequency of hyperglycaemia and greater satisfaction with continuing treatment in the QW group compared with the BID group. Effect sizes for change in DTSQ-s total scores were 0.84 QW, 0.64 BID; for IWQOL-Lite: 0.96 QW, 0.82 BID. Treatment satisfaction and QOL improved significantly between weeks 30 and 52 for those switching from BID to QW. Occurrence of adverse events did not affect patients’ improvements in treatment satisfaction and QOL.

**Conclusions:**

Patients treated with exenatide QW or BID experienced significant and clinically meaningful improvements in treatment satisfaction and QOL. Patients who switched from exenatide BID to exenatide QW administration reported further significant improvements.

## Introduction

The incidence of Type 2 diabetes is increasing [1]. Over 80% of all people with Type 2 diabetes are overweight and over 50% are obese [2]. For patients with diabetes, obesity exacerbates metabolic problems, leading to increased morbidity and mortality [3]. Both Type 2 diabetes [4] and obesity [5] are associated with diminished health-related quality of life (QOL). Unfortunately, some effective glucose-lowering therapies may not only contribute to weight gain [6], but they may also lower treatment satisfaction and QOL [7–9].

Exenatide is a first-in-class glucagon-like peptide 1 receptor agonist approved for the treatment of Type 2 diabetes. Exenatide improves glycaemic and clinical parameters that could affect treatment satisfaction and QOL, including improved blood glucose control, decreased appetite and reduced weight [10,11]. Significant benefits have been seen in patients taking exenatide twice daily (BID) and in those taking an exenatide once weekly (QW) formulation [10–12].

The current study is designed to assess the effects of exenatide BID and QW treatment on treatment satisfaction and QOL of patients with Type 2 diabetes managed with diet and exercise and/or oral glucose-lowering medications [13,14]. Treatment satisfaction deserves attention because it may influence treatment adherence [15] and consequent clinical outcomes [15–17]. Quality of life is a critical outcome in its own right and a growing number of clinical trials now incorporate measures of health-related QOL as primary or secondary outcomes. We chose to use disease-specific measures for treatment satisfaction and QOL in the current study because such measures are generally considered more sensitive than generic measures to the predicted effects of clinical trial interventions [18].

In this study we attempted to answer the following questions: (i) does exenatide treatment affect diabetes treatment satisfaction and are changes in treatment satisfaction different for patients taking exenatide QW and BID?; (ii) does exenatide treatment affect weight-related QOL and are changes in weight-related QOL different for patients taking exenatide QW and BID?; (iii) do patients who switch from exenatide BID to exenatide QW experience further improvement in treatment satisfaction and QOL. In addition, we also assessed whether there are any differences in treatment satisfaction and weight-related QOL in those patients who did or did not report certain adverse events (AEs).

## Research design and methods

### Data source

Data for this study were obtained from a randomized, multi-centre, open-label study of subjects with Type 2 diabetes managed with diet and exercise and/or oral glucose-lowering medications. Two hundred and ninety-five patients received exenatide either once weekly (2 mg, QW) or twice daily (10 μg, BID) during weeks 1–30. The primary endpoint of the study was change in glycated haemoglobin (HbA_1c_) at 30 weeks. Following 30 weeks, 258 patients continued on to an open-ended treatment period with exenatide QW. Results of this study to 30 weeks and to 52 weeks for HbA_1c_, fasting plasma glucose, weight and adverse events are reported elsewhere [13,14]. Briefly, the exenatide QW group showed greater improvements in HbA_1c_ (−1.9 vs. −1.5% for exenatide BID at 30 weeks; *P* = 0.0023) and fasting plasma glucose (−2.3 vs. −1.4 mmol/l for exenatide BID; *P* < 0.0001), with similar levels of weight loss and adverse events. There was no major hypoglycaemia in either exenatide regimen. One patient who received exenatide QW with non-sulphonylurea background therapy had an episode of minor hypoglycaemia. Most minor hypoglycaemia occurred in patients using concomitant sulphonylurea (eight of 55 receiving exenatide QW and eight of 54 receiving exenatide BID).

The presence of an AE of nausea or vomiting was defined as having at least one treatment-emergent adverse event (TEAE) during the 30-week treatment period; two subjects in each treatment arm withdrew from the study as a result of nausea or vomiting. The presence of injection site reactions was defined as having at least one TEAE during the 30-week comparison period with Medical Dictionary for Regulatory Activities (MedDRA) preferred terms comprising any of the following ‘injection site’ terms: bruising, erythema, haematoma, haemorrhage, induration, irritation, nodule, pain, pruritus, rash, swelling or urticaria.

### Patient-reported outcome (PRO) instruments

Patients completed a measure of diabetes treatment satisfaction, the Diabetes Treatment Satisfaction Questionnaire status version (DTSQ-s) and a measure of QOL, the Impact of Weight on Quality of Life—Lite (IWQOL-Lite), at baseline and weeks 30 and 52. Patients were asked to complete the PRO instruments at the beginning of their clinic visit, prior to any procedures. Patients who terminated their participation prior to week 30 were asked to complete the study questionnaires as part of their early termination assessment.

### Diabetes Treatment Satisfaction Questionnaire (DTSQ)

Diabetes treatment satisfaction was assessed with the Diabetes Treatment Satisfaction Questionnaire—status version (DTSQ-s) [19].

The DTSQ-s contains eight items assessing: overall treatment satisfaction, treatment convenience and flexibility, satisfaction with understanding of diabetes, willingness to continue present treatment and to recommend it to others and frequency of unacceptably high and unacceptably low blood glucose levels. Response categories for all items are on a 7-point Likert scale. DTSQ-s scores range from 0 (e.g. very dissatisfied) to 6 (e.g. very satisfied). All items except perceived hypoglycaemia and hyperglycaemia items are summed to produce a total treatment satisfaction score. The DTSQ-s total treatment satisfaction scores range from 0 to 36. Higher scores on the DTSQ-s total score indicate higher satisfaction. The perceived frequency of hyperglycaemia and hypoglycaemia items are scored separately; lower scores on these two items represent better perceived blood glucose control. Missing data at each assessment were imputed as the average of the valid item values.

### Weight-related quality of life (IWQOL-Lite)

The impact of weight-related QOL was assessed with the IWQOL-Lite, a 31-item self-report measure assessing weight-related QOL in five domains: physical function, self-esteem, sexual life, public distress and work [5]. The IWQOL-Lite has demonstrated robust psychometric properties in obese persons with and without diabetes [20]. IQWOL-Lite scores (total score and separate scores for each of the five domains) range from 0 to 100, with 0 representing the worst outcome and 100 representing the best. Raw scores for each of the IWQOL-Lite scales were computed only if at least 50% of the items for that scale were answered, and the total score was computed only if at least 75% of all items were answered. Missing data at each follow-up assessment were imputed as the average of the valid item values.

### Statistical analysis

The intent-to-treat population, defined as all randomized subjects who received at least one injection of study medication (exenatide), was used. All tests of treatment effects were conducted at a two-sided significance level of 0.05. There was no adjustment in significance levels for multiple comparisons. The pre-specified primary analysis of PROs was to compare the treatment effects between groups at week 30.

ancova [adjusted for baseline DTSQ-s or IWQOL-Lite score, HbA_1c_ strata (screening HbA_1c_ < 9.0 vs. ≥ 9.0%), treatment group and sulphonylurea use at screening (Yes vs. No)] was used to estimate least squares (LS) mean group changes from baseline to week 30. Two-sided 95% confidence intervals (CIs) were computed for changes at week 30. ancova used last observation carried forward (LOCF); the last available post-baseline observation (including Early Termination) was used to impute missing data for the PRO measures, as long as the subject had PRO data for at least one post-baseline (including Early Termination) visit.

LS mean change and the within-group standard deviation of change were used to calculate standard response mean (SRM) effect sizes (i.e. measure the magnitude of treatment effect for each group) [21]. Effect sizes of small, medium and large are indicated by cut-offs of 0.20, 0.50 and 0.80, respectively [21,22].

Pairwise Student’s*t*-tests for correlated outcomes were used to estimate change in DTSQ-s and IWQOL-Lite from week 30 to week 52 for each treatment group. Change within the group switching from exenatide BID to QW provides a direct test of the effect of changing exenatide regimens.

## Results

### Baseline characteristics

The ITT population comprised 295 patients (148 exenatide QW, 147 exenatide BID). Demographic and baseline clinical variables are summarized in Table 1. Mean age of study participants was 55 years, slightly more than half were male and the majority of the participants were white, with a mean body mass index (BMI) of 35 kg/m^2^, a mean HbA_1c_ of 8.3% and mean diabetes duration of 6–7 years. On entry to the study, participants were treated either with diet and exercise alone or with one or more of a variety of oral glucose-lowering agents. There were no statistically significant differences between the two treatment groups for any of these characteristics [13] nor for baseline PRO scores.

**Table 1:** Baseline demographic and clinical characteristics of subjects with Type 2 diabetes participating in a 30-week, randomized, multi-centre, open-label study of exenatide treatment

Characteristic	Exenatide QW (*n* = 148)	Exenatide BID (*n* = 147)
Age (years), mean ± sd	55 ± 10	55 ± 10
Gender, *n* (%)		
Male	82 (55)	75 (51)
Female	66 (45)	72 (49)
Race/ethnicity, *n* (%)		
White	123 (83)	107 (73)
Black	9 (6)	19 (13)
Hispanic	16 (11)	20 (14)
Asian	0 (0)	1 (1)
Weight (kg), mean ± sd	102 ± 19	102 ± 21
BMI (kg/m^2^), mean ± sd	35 ± 5	35 ± 5
HbA_1c_ (%), mean ± sd	8.3 ± 1.0	8.3 ± 1.0
Duration of diabetes (years), mean ± sd	7 ± 6	6 ± 5
Diabetes management at screening, *n* (%)		
Diet/exercise	21 (14)	23 (16)
Metformin only	56 (38)	50 (34)
Sulphonylureas only	6 (4)	10 (7)
Thiazolidinediones only	2 (1)	7 (5)
Metformin + sulphonylureas	43 (29)	39 (27)
Metformin + thiazolidinediones	13 (9)	11 (8)
All metformin*	114 (77)	102 (69)
All sulphonylureas*	55 (37)	54 (37)
All thiazolidinediones*	22 (15)	25 (17)

* Includes specified agent alone or in combination.

BID, twice daily; BMI, body mass index; HbA_1c_, glycated haemoglobin; QW, once weekly; sd, standard deviation.

### Effect of exenatide treatment on diabetes treatment satisfaction

At week 30, total DTSQ-s scores had improved significantly from baseline in both treatment arms (*P* < 0.001), with significant improvement for all specific items, except for perceived hypoglycaemia frequency in the exenatide QW arm, and significant improvement for all items, except perceived hypoglycaemia frequency and treatment convenience in the exenatide BID arm. Effect sizes for change in DTSQ-s total scores at week 30 were 0.84 for exenatide QW compared with 0.64 for exenatide BID. Statistically significant improvements in total treatment satisfaction for both treatment regimens met the conventional criterion for clinical meaningfulness [≥ 0.5 standard deviation (sd) units], as assessed by the standardized response mean [22]. Effect sizes for change in DTSQ-s individual items from baseline to week 30 ranged from 0.06 to 1.25 in the exenatide QW group and 0.11 to 0.87 in the exenatide BID group.

At week 30, between-treatment group differences in DTSQ-s total scores were not statistically significant (*P* = 0.09), but treatment satisfaction did improve more in the exenatide QW arm for perceived hyperglycaemia frequency (*P* = 0.03) and willingness to continue current treatment (*P* = 0.01).

From week 30 to week 52 (Table 3), patients who switched from exenatide BID to exenatide QW experienced significantly improved total treatment satisfaction (*P* = 0.037), treatment convenience (*P* = 0.003), treatment flexibility (*P* = 0.012) and satisfaction with continuing treatment (*P* = 0.048). Patients who continued with exenatide QW experienced significantly improved satisfaction with treatment convenience (*P* = 0.006) and treatment flexibility (*P* = 0.025) from week 30 to week 52. All significant improvements in treatment satisfaction from baseline to week 30 remained significant in comparisons from baseline to week 52.

**Table 3:** Change (± sd) from week 30 to week 52 in Diabetes Treatment Satisfaction Questionnaire—Status version (DTSQ-s) and Impact of Weight on Quality of Life—Lite (IWQOL-Lite) among subjects with Type 2 diabetes participating in a randomized, open-label study of exenatide treatment (intent-to-treat population)

	Exenatide QW		Exenatide BID → QW	
	*n*†	Change	*n*†	Change
DTSQ				
DTSQs total score	119	0.65 ± 3.8	123	1.16 ± 6.1*
Treatment satisfaction—current	119	0.10 ± 0.77	123	0.01 ± 1.2
Perceived frequency high blood sugar	119	−0.04 ± 1.9	123	−0.15 ± 1.8
Perceived frequency low blood sugar	119	−0.18 ± 1.4	123	−0.21 ± 1.4
Treatment convenience	119	0.30 ± 1.2**	123	0.42 ± 1.6**
Treatment flexibility	119	0.24 ± 1.2*	122	0.39 ± 1.7*
Understanding of diabetes	119	0.06 ± 0.94	123	0.04 ± 1.2
Treatment recommend	119	−0.05 ± 0.94	123	0.07 ± 1.1
Treatment satisfaction—continue	119	−0.01 ± 0.93	123	0.24 ± 1.3*
IWQOL-Lite				
IWQOL-Lite total score	120	0.36 ± 7.1	127	1.44 ± 8.7
Physical function	121	0.08 ± 9.5	128	2.13 ± 11.5*
Self-esteem	121	0.83 ± 10.9	128	1.12 ± 11.5
Sexual life	118	0.64 ± 17.3	124	0.91 ± 15.2
Public distress	120	6.96 ± 13.2***	127	5.04 ± 11.2***
Work	119	0.42 ± 9.8	125	1.10 ± 11.2

* *P* ≤ 0.05;

** *P* < 0.01;

*** *P* < 0.001.

† Number of subjects with week 30 and week 52 scores.

BID, twice daily; QW, once weekly; sd, standard deviation.

### Effects of exenatide treatment on weight-related quality of life

At week 30 (Table 2), IWQOL-Lite total scores and all separate domain scores had increased significantly in both treatment arms (all *P* < 0.001). Effect sizes for change in IWQOL-Lite total score at week 30 were 0.96 for the exenatide QW and 0.82 for the exenatide BID group. Statistically significant improvements in total treatment satisfaction for both treatment regimens met the conventional criterion for clinical meaningfulness (≥ 0.5 sd units), as assessed by the standardized response mean [21,22]. There were no statistically significant differences in weight-related QOL between treatment arms.

**Table 2:** Change (± se)† from baseline to week 30 in Diabetes Treatment Satisfaction Questionnaire—Status version (DTSQ-s) and Impact of Weight on Quality of Life—Lite (IWQOL-Lite) among subjects with Type 2 diabetes participating in a randomized, multi-centre, open-label study of exenatide treatment (intent-to-treat population)

	Exenatide QW					Exenatide BID					
Patient reported-outcomes	*n*‡	Endpoint	Change	95% CI	Effect size§	*n*‡	Endpoint	Change	95% CI	Effect size§	*P*-value¶
DTSQ											
DTSQ-s total score	137	31.17	5.17 ± 0.54***	4.11, 6.23	0.84	142	29.97	3.97 ± 0.53***	2.94, 5.01	0.64	0.09
Treatment satisfaction—current	130	5.29	1.29 ± 0.12***	1.06, 1.52	0.94	136	5.11	1.11 ± 0.11***	0.89, 1.33	0.87	0.23
Perceived frequency high blood sugar	130	1.95	−1.86 ± 0.15***	−2.16, −1.55	1.09	136	2.39	−1.42 ± 0.15***	−1.71, −1.12	0.81	0.03
Perceived frequency low blood sugar	131	0.92	0.08 ± 0.12	−0.15, 0.31	0.06	136	0.98	0.14 ± 0.11	−0.08, 0.37	0.11	0.69
Treatment convenience	130	4.79	0.25 ± 0.13*	0.00, 0.50	0.17	136	4.68	0.14 ± 0.13	−0.11, 0.39	0.09	0.52
Treatment flexibility	130	4.91	0.49 ± 0.13***	0.23, 0.75	0.33	135	4.69	0.27 ± 0.13*	0.01, 0.52	0.18	0.19
Understanding of diabetes	132	4.95	0.50 ± 0.10***	0.29, 0.70	0.44	136	5.02	0.56 ± 0.10***	0.36, 0.76	0.48	0.63
Treatment recommend	130	5.59	0.98 ± 0.10***	0.79, 1.17	0.86	136	5.44	0.83 ± 0.10***	0.64, 1.01	0.71	0.23
Treatment satisfaction—continue	130	5.46	1.43 ± 0.13***	1.18, 1.68	1.25	136	5.02	0.99 ± 0.12***	0.75, 1.24	0.85	0.01
IWQOL-Lite											
IWQOL-Lite total score	143	82.74	10.23 ± 0.89***	8.48, 11.97	0.96	143	81.13	8.61 ± 0.88***	6.88, 10.34	0.82	0.16
Physical function	126	77.72	12.06 ± 1.20***	9.67, 14.45	0.89	130	75.21	9.55 ± 1.21***	7.17, 11.92	0.69	0.11
Self-esteem	126	80.47	13.60 ± 1.31***	11.02, 16.18	0.83	130	80.58	13.71 ± 1.30***	11.14, 16.28	0.85	0.95
Sexual life	123	84.09	7.34 ± 1.51***	4.36, 10.32	0.44	127	83.7	6.95 ± 1.50***	4.00, 9.89	0.41	0.84
Public distress	125	89.83	6.05 ± 1.00***	4.07, 8.02	0.52	130	87.85	4.07 ± 1.00***	2.11, 6.03	0.36	0.13
Work	124	91.14	8.17 ± 1.13***	5.95, 10.38	0.65	129	88.79	5.81 ± 1.12***	3.61, 8.01	0.46	0.11

* *P* ≤ 0.05;

*** *P* < 0.001.

† Data are least squares (LS) mean changes.

‡ Number of subjects with a post-randomization score.

§ Standardized response mean.

¶ Comparisons between treatment groups.

BID, twice daily; CI, confidence interval; QW, once weekly; se, standard error.

Patients who switched from exenatide BID to QW at week 30 (Table 3) reported further significant improvement for the physical function (*P* = 0.04) and public distress (*P* < 0.001) domains. Patients who continued on QW improved significantly from week 30 to week 52 for public distress (*P* < 0.001). All significant improvements in QOL from baseline to week 30 remained significant in comparisons from baseline to week 52.

### Potential mediators/moderators of effects on treatment satisfaction and quality of life

There was no significant difference in total treatment satisfaction in the 89 subjects who experienced nausea and/or vomiting vs. the 190 who did not (*P* = 0.97) and the effect did not differ across treatment arms (*P* = 0.59). Treatment satisfaction was also similar for patients who did (*n* = 61) or did not (*n* = 218) experience an injection site reaction (*P* = 0.41); the effect did not differ across treatment arms (*P* = 0.44).

There was no significant difference in overall weight-related QOL in subjects who did and did not experience nausea (*P* = 0.56), a common side effect of exenatide therapy, and the effect did not differ across treatment arms (*P* = 0.42). There was also no significant difference in weight-related quality of life in subjects who did and did not experience an injection site reaction (*P* = 0.70); the effect did not differ across treatment arms (*P* = 0.49).

## Discussion

In this study, treatment satisfaction and weight-related QOL were significantly improved with the addition of exenatide to treatment with diet, exercise and/or oral glucose-lowering medication. Benefits were substantial and were manifest across the full range of dimensions studied. Treatment satisfaction and weight-related QOL improved significantly from baseline to week 30 in both treatment arms, with no significant difference between treatment arms in total treatment satisfaction or QOL, but a greater improvement in the exenatide QW arm in perceived hyperglycaemia frequency and willingness to continue current treatment. All improvements were sustained from week 30 to week 52 among patients who continued with exenatide QW; moreover, there were significant improvements in treatment convenience and treatment flexibility. In addition, patients who switched from exenatide BID to exenatide QW at week 30 reported significantly improved total treatment satisfaction, treatment convenience, treatment flexibility and satisfaction with continuing treatment at week 52.

This study is the first to assess treatment satisfaction and quality of life in patients treated with exenatide QW and our findings suggest that both exenatide QW and exenatide BID are associated with statistically significant and clinically meaningful improvements (i.e. moderate or greater effect sizes) in these important patient-reported outcomes. The fact that improvements in treatment satisfaction and quality of life were maintained over 52 weeks suggests that these effects are durable and the fact that participants continued to prefer exenatide to their pre-study treatment regimen suggests that patients may be willing to continue to manage their Type 2 diabetes with exenatide treatment. Sustained use in the general population of patients with diabetes could bring many patients the benefits associated with exenatide treatment in clinical trials, including improved blood glucose control and significantly reduced weight [10–14].

We found some evidence that use of exenatide QW was associated with greater improvements in treatment satisfaction than use of exenatide BID. At week 30, patients in the exenatide QW arm were significantly more likely than those in the exenatide BID arm to be willing to continue taking the study medication and, between weeks 30 and 52, patients who switched to exenatide QW improved on a number of treatment satisfaction measures, including total satisfaction and willingness to continue treatment. This suggests that acceptance of exenatide QW may be even greater than that for exenatide BID, perhaps because of ease of use and less frequent administration regimen of once weekly. Another possible reason for the greater acceptance of exenatide QW is the greater improvement in glucose control [13,14]; notably, reduction in the perceived frequency of hyperglycaemia was the specific benefit for which exenatide QW had the largest advantage over exenatide BID.

The common adverse effects of treatment experienced in this study (nausea/vomiting or injection site reactions), which were more common in the exenatide BID group [13,14], did not affect patients’ treatment satisfaction or quality of life. This suggests that these adverse effects were not severe enough to affect patients’ perceptions of the study medications.

### Study strengths

Strengths of the study include the substantial number of participants and the relatively long duration of the trial. In addition, the study outcomes, treatment satisfaction and quality of life were assessed using validated questionnaires likely to be sensitive to the established clinical effects of the study medication. The study design allowed us to assess not only the effects of each formulation of the study medication in patients who had never taken exenatide, but also the effects of switching to exenatide QW in patients who had been taking exenatide BID. Finally, we were able to assess the effects of commonly reported medication side effects on treatment satisfaction and quality of life.

### Study limitations

Study design limitations include the fact that there was no placebo comparator group that continued with their pre-study medications to assess placebo effects and there was no crossover from exenatide QW to exenatide BID to assess order effects. These features would have required a larger and longer-term study. A longer-term follow-up of exenatide QW would have been beneficial, but the design did permit 52 weeks of patient experience which is the longest term study of exenatide QW to date; 3.5-year follow-up data on exenatide BID has already been published [23]. Also, the participation of ethnic minorities was rather low, limiting the generalizability of the study findings to these groups. Finally, it would have been useful to examine outcomes for psychological well-being and diabetes distress.

### Implications for future research

While the results suggest that exenatide BID and QW are both viable treatment strategies, it remains to be seen whether the different medications are preferred by different patient subgroups. Systematic evaluation of patient differences that account for alternative preferences should be pursued.

### Clinical implications

Exenatide treatment has been associated with important clinical benefits, including improved glucose control and weight loss [10,11,13,14]. Patients previously treated with diet and exercise and/or oral medication reported improved weight-related QOL, higher satisfaction with the study medication than their previous therapy and more willingness to continue taking the medication and recommend it to others. They also reported that the study medication was more flexible and convenient, despite the fact that it involved taking injections. Moreover, the recognized side effects of exenatide treatment—nausea/vomiting and injection site reactions—did not affect treatment satisfaction and quality of life in this study, suggesting that these effects might not be a barrier to patients’ accepting treatment with these medications.

Finally, patients switching from exenatide BID to exenatide QW reported additional improvements in treatment convenience and overall treatment satisfaction. Not surprisingly, patients seem to prefer taking a medication once a week rather than twice a day. This simpler regimen could improve treatment adherence in real-world clinical settings [24].

In combination with earlier findings from this study [13,14], our results indicate it is possible for patients treated with diet/exercise and/or oral agents to initiate exenatide therapy with potential benefits in both clinical efficacy and patient-reported outcomes directly related to treatment adherence.

## Abbreviations

AE: adverse event
BID: twice daily
DTSQ: Diabetes Treatment Satisfaction Questionnaire
HbA_1c_: glycated haemoglobin
IWQOL-Lite: Impact of Weight on Quality of Life—Lite
LS: least squares
PRO: patient-reported outcome
QOL: quality of life
QW: once weekly
sd: standard deviation
TEAE: treatment-emergent adverse event

## References

[1] Mokdad AH, Ford ES, Bowman BA, Dietz WH, Vinocor F, Bales VS (2003). Prevalence of obesity, diabetes, and obesity-related health risk factors. J Am Med Assoc.

[2] Bays HE, Chapman RH, Grandy S, for the SHIELD Investigators’ Group (2007). The relationship of body mass index to diabetes mellitus, hypertension and dyslipidaemia: comparison of data from two national surveys. Int J Clin Pract.

[3] Maggio CA, Pi-Sunyer FX (1997). The prevention and treatment of obesity: application to type 2 diabetes. Diabetes Care.

[4] Redekkop WK, Koopmanschap MA, Stolk RP, Rutten GEH, Niessen LW (2002). Health-related quality of life and treatment satisfaction in Dutch patients with type 2 diabetes. Diabetes Care.

[5] Kolotkin RL, Crosby RD, Kosloski KD, Williams GR (2001). Development of a brief measure to assess quality of life in obesity. Obes Res.

[6] UK Prospective Diabetes Study Group (1998). Intensive blood-glucose control with sulphonylurea or insulin compared with conventional treatment and risk of complications in patients with type 2 diabetes (UKPDS 33). Lancet.

[7] Jacobson AM, de Groot M, Samson JA (1994). The evaluation of two measures of quality of life in patients with type 1 and type 2 diabetes. Diabetes Care.

[8] Glasgow RE, Ruggiero L, Eakin EG, Dryfoos J, Chobanian L (1997). Quality of life and associated characteristics in a large national sample of adults with diabetes. Diabetes Care.

[9] Van der Does FE, De Neeling JN, Snoek FJ, Grootenhuis PA, Kostense PJ, Bouter LM (1998). Randomized study of two different levels of glycemic control within the acceptable range in type 2 diabetes. Diabetes Care.

[10] Defronzo RA, Okerson T, Viswanathan P, Guan X, Holcombe JH, MacConell L (2008). Effects of exenatide versus sitagliptin on postprandial glucose, insulin and glucagon secretion, gastric emptying, and caloric intake: a randomized, cross-over study. Curr Med Res Opin.

[11] Kim D, MacConell L, Zhuang D, Kothare PA, Trautmann M, Fineman M (2007). Effects of once-weekly dosing of a long-acting release formulation of exenatide on glucose control and body weight in subjects with type 2 diabetes. Diabetes Care.

[12] Kendall DM, Riddle MC, Rosenstock J, Zhuang D, Kim DD, Fineman MS (2005). Effects of exenatide (Extendin-4) on glycemic control over 30 weeks in patents with type 2 diabetes treated with metformin and a sulfonylurea. Diabetes Care.

[13] Drucker DJ, Buse JB, Taylor K, Kendall DM, Trautmann M, Zhuang D, for the DURATION-1 Study Group (2008). Exenatide once weekly versus twice daily for the treatment of type 2 diabetes: a randomized, open-label, non-inferiority study. Lancet.

[14] Buse J, Drucker D, Taylor K, Kim T, Wilhelm K, Kendall D (2008). Exenatide once weekly elicits sustained glycaemic control and weight loss over 52 weeks. Diabetologia.

[15] Charpentier G, Fleury F, Dubroca I, Vaur L, Clerson P (2005). Electronic pill-boxes in the evaluation of oral hypoglycemic agent compliance. Diabetes Metab.

[16] Kelley K, Dempsey C (2007). An evaluation of an insulin transfer programme delivered in a group setting. J Clin Nurs.

[17] Tahrani AA, Digwood S, Lee C, Moulilk P (2007). Evaluation of glargine group-start sessions in patients with type 2 diabetes as a strategy to deliver the service. Int J Clin Pract.

[18] Leidy NK, Revicki DA, Geneste B (1999). Recommendations for evaluating the validity of quality of life claims for labeling and promotion. Value Health.

[19] Bradley C, Chur BC (1994). The Diabetes Treatment Satisfaction Questionnaire: DTSQ. Handbook of Psychology and Diabetes: A Guide to Psychological Measurement in Diabetes Research and Practice.

[20] Kolotkin RL, Crosby RD, Williams GR (2003). Assessing weight-related quality of life in obese persons with type 2 diabetes. Diabetes Res Clin Pract.

[21] Norman GR, Wyrwich KW, Patrick DL (2007). The mathematical relationship among different forms of responsiveness coefficients. Qual Life Res.

[22] Norman GR, Sloan JA, Wyrwich KW (2003). Interpretation of changes in health-related quality of life: the remarkable universality of half a standard deviation. Med Care.

[23] Klonoff KC, Buse JB, Nielson LL, Guan X, Bowlus CL, Holcombe JH (2008). Exenatide effects on diabetes, obesity, cardiovascular risk factors and hepatic biomarkers in patients with type 2 diabetes treated for at least 3 years. Curr Med Res Opin.

[24] Rubin RR (2005). Adherence to pharmacologic therapy in patients with type 2 diabetes mellitus. Am J Med.

